# Heart–lung interactions during neurally adjusted ventilatory assist

**DOI:** 10.1186/s13054-014-0499-8

**Published:** 2014-09-12

**Authors:** David Berger, Stefan Bloechlinger, Jukka Takala, Christer Sinderby, Lukas Brander

**Affiliations:** Department of Intensive Care Medicine, Bern University Hospital (Inselspital) and University of Bern, 3010 Bern, Switzerland; Department of Cardiology, Bern University Hospital (Inselspital) and University of Bern, Bern, Switzerland; Department of Medicine and Interdepartmental Division of Critical Care Medicine, University of Toronto, Suite RFE 3-805, 200 Elizabeth Street, Toronto, ON Canada M5G 2C4; Keenan Research Centre for Biomedical Science of St. Michael’s Hospital; Department of Critical Care, St. Michael’s Hospital, 30 Bond Street, Toronto, Ontario Canada M5B1W8; Department of Anesthesia and Intensive Care Medicine, Luzerner Kantonsspital, 6000 Luzern 16, Switzerland

## Abstract

**Introduction:**

Assist in unison to the patient’s inspiratory neural effort and feedback-controlled limitation of lung distension with neurally adjusted ventilatory assist (NAVA) may reduce the negative effects of mechanical ventilation on right ventricular function.

**Methods:**

Heart–lung interaction was evaluated in 10 intubated patients with impaired cardiac function using esophageal balloons, pulmonary artery catheters and echocardiography. Adequate NAVA level identified by a titration procedure to breathing pattern (NAVAal), 50% NAVAal, and 200% NAVAal and adequate pressure support (PSVal, defined clinically), 50% PSVal, and 150% PSVal were implemented at constant positive end-expiratory pressure for 20 minutes each.

**Results:**

NAVAal was 3.1 ± 1.1cmH_2_O/μV and PSVal was 17 ± 2 cmH_2_0. For all NAVA levels negative esophageal pressure deflections were observed during inspiration whereas this pattern was reversed during PSVal and PSVhigh. As compared to expiration, inspiratory right ventricular outflow tract velocity time integral (surrogating stroke volume) was 103 ± 4%, 109 ± 5%, and 100 ± 4% for NAVAlow, NAVAal, and NAVAhigh and 101 ± 3%, 89 ± 6%, and 83 ± 9% for PSVlow, PSVal, and PSVhigh, respectively (p < 0.001 level-mode interaction, ANOVA). Right ventricular systolic isovolumetric pressure increased from 11.0 ± 4.6 mmHg at PSVlow to 14.0 ± 4.6 mmHg at PSVhigh but remained unchanged (11.5 ± 4.7 mmHg (NAVAlow) and 10.8 ± 4.2 mmHg (NAVAhigh), level-mode interaction p = 0.005). Both indicate progressive right ventricular outflow impedance with increasing pressure support ventilation (PSV), but no change with increasing NAVA level.

**Conclusions:**

Right ventricular performance is less impaired during NAVA compared to PSV as used in this study. Proposed mechanisms are preservation of cyclic intrathoracic pressure changes characteristic of spontaneous breathing and limitation of right-ventricular outflow impedance during inspiration, regardless of the NAVA level.

**Trial registration:**

Clinicaltrials.gov Identifier: NCT00647361, registered 19 March 2008

**Electronic supplementary material:**

The online version of this article (doi:10.1186/s13054-014-0499-8) contains supplementary material, which is available to authorized users.

## Introduction

Cyclic increases in intrathoracic pressure during positive pressure ventilation may reduce venous return and increase the afterload of the right ventricle [1,2]. Neurally adjusted ventilatory assist (NAVA) delivers inspiratory support in synchrony and in linear proportion to the neural inspiratory effort by using the electrical activity of the diaphragm (EAdi) to drive the ventilator [3]. The inspiratory muscle activity, that is synchronous and proportional to assist delivery during NAVA, is likely to attenuate the increases in pleural pressure associated with conventional positive pressure ventilation. This attenuation should reduce negative effects of positive pressure ventilation on cardiovascular function. Data on cardiovascular function during NAVA are very limited. The cardiovascular effects of NAVA depend on the pleural pressure changes and the consequent transmural vascular and cardiac pressures, but these have not so far been studied.

We hypothesized that the synchronous inspiratory muscle activity during NAVA and the feedback controlled limitation of lung volumes during increasing NAVA support avoid the adverse effects of conventional pressure support ventilation (PSV) on right ventricular afterload and venous return. We therefore compared the short-term hemodynamic effects of three NAVA and three PSV levels using simultaneous analysis of intravascular, intracardiac, esophageal pressure (Pes), airway pressure, breathing pattern, and echocardiography in patients with impaired cardiac function, chronic obstructive pulmonary disease, or both.

## Materials and methods

The Ethics Committee of Canton Bern, Switzerland approved the protocol (KEK Nr 217–06). Recruitment lasted from March to November 2010. Written informed consent from the patient’s family and deferred consent after recovery were obtained. A detailed description of the methods is provided in Additional file 1.

The main inclusion criteria were invasive mechanical ventilation, pneumatic triggering, pulmonary artery catheter monitoring for clinical reasons, and at least one of the following: left ventricular ejection fraction ≤40%; inotropic drugs (dobutamine ≥2 μg/kg/minute or adrenaline ≥0.03 μg/kg/minute); pulmonary artery occlusion pressure ≥18 mmHg; or history of chronic obstructive pulmonary disease.

### Study protocol

**Figure 1:**
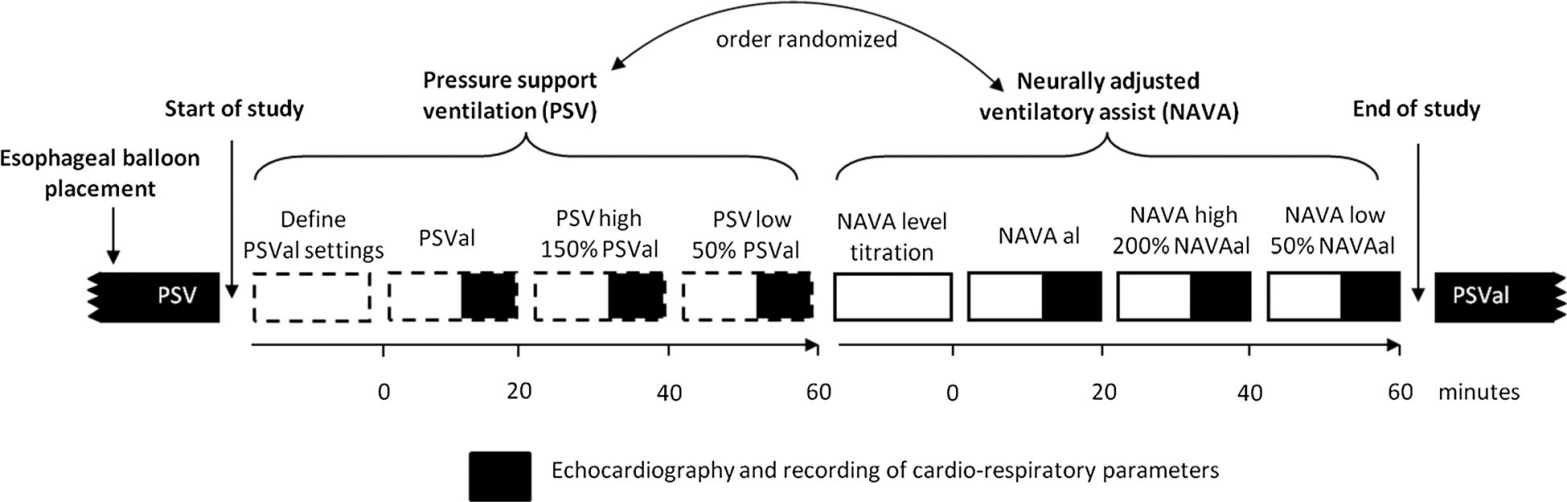
**Schematic study protocol.** After a baseline period of 30 minutes three levels of pressure support ventilation (PSV) and three levels of neurally adjusted ventilatory assist (NAVA) were applied in random order. Adequate PSV (PSVal) was chosen by an independent intensivist using predefined clinical criteria, and adequate NAVA (NAVAal) using a titration procedure [6-9]. NAVAhigh, highest level with constant breathing pattern or a maximum 200% of NAVAal; NAVAlow, 50% of NAVAal; PSVhigh, 150% of PSVal; PSVlow, 50% of PSVal.

Catheters were inserted for measurement of Pes (SmartCath; Viasys Healthcare, San Diego, CA, USA) and EAdi (Maquet, Solna, Sweden). Correct positioning of the Pes catheter was verified using an occlusion test [4,5]. A schematic study protocol is depicted in Figure 1.

Adequate PSV (PSVal) was set by an independent intensivist blinded for the EAdi and Pes aiming to achieve optimal patient comfort, avoid unassisted wasted inspiratory efforts, and minimize negative inspiratory deflections in central venous and pulmonary artery pressures (indicating inspiratory effort). PSVlow was 50% PSVal, and PSVhigh was 150% PSVal. Positive end-expiratory pressure (PEEP) and other prescribed ventilator settings were kept constant throughout the protocol.

Adequate NAVA (NAVAal) was identified using a previously described titration procedure (Additional files 1 and 2 [6-9]). NAVAlow was 50% NAVAal, and NAVAhigh was defined as the highest level sustaining a regular breathing pattern similar to that at NAVAal or 200% NAVAal, whichever occurred first.

The order of the ventilatory modes was randomized. The six ventilator settings were applied for at least 20 minutes each. All medications including sedation (Richmond agitation and sedation scale of −1 to −2 [10]) remained unchanged.

### Measurements

Intravascular pressures, airway pressure, Pes, EAdi (as the percentage of EAdi without assist at the beginning of NAVA titration), and airflow were recorded continuously (Neurovent Research Inc., Toronto, ON, Canada). Pulmonary artery occlusion pressure, right ventricular pressure, and blood gases were measured at the end of each experimental period.

All signals were analyzed off-line breath by breath, using a semi-automated detection of inspiration and expiration based on airflow reversal [6-8]. Two approaches were used to evaluate the hemodynamic responses. First, intravascular pressure and Pes during all breaths in each ventilator setting were averaged separately during inspiration and expiration. This was done in order to include the impact of variability in breathing pattern on hemodynamics. Transmural vascular pressures were obtained by subtraction of the time integral of Pes from the respective intravascular pressure. Changes of intravascular pressures from inspiration to expiration (cyclic pressure changes) were characterized by subtracting the mean expiratory pressure from the mean inspiratory pressure (Additional file 3). The mean inspiratory Pes deflection was calculated for the duration of inspiratory airflow and was referenced to the Pes immediately preceding the start of inspiratory airflow.

Second, representative single-breath cycles were manually selected for each experimental period. The selected breaths had mean inspiratory transpulmonary pressures equal to the mean of all breaths for the specific study period (the average breath). For each of these single breaths, the central venous pressure (CVP) was measured at the base of the c wave [11]. The right ventricular isovolumetric pressure change was calculated as the difference between the base of the c wave of CVP and the pulmonary artery pressure (PAP) at valve opening [12], and the right ventricular total pressure generation was calculated as the pressure difference from the end-diastolic filling of the right ventricle (estimated as the base of the CVP c wave) to the systolic PAP. An example of this analysis is given in Additional file 4.

Pulsed wave Doppler profiles from both ventricular outflow tracts were recorded simultaneously with airway pressure during transthoracic echocardiography and were analyzed offline (Vivid 7, EchoPAC Dimension ‘06; GE Medical Systems, Glattbrugg, Switzerland). End-inspiratory and end-expiratory heart beats [13] from three ventilatory cycles were analyzed for each condition. To characterize the effects of lung inflation on the Doppler flow profiles, the values at end inspiration were expressed as a percentage of the values at end expiration measured during the same breathing cycle. A value >100% would thus reflect a higher value during inspiration compared with expiration, and *vice versa*.

### Statistical analysis

Repeated-measures analysis of variance (within-subject factors: ventilation mode, support level) was used for analysis. Significant mode*support level interactions were analyzed *post hoc* within each mode between support levels using Sidak’s correction (IBM SPSS 20.0.0; IBM Corp, Armonk, NY, USA). Data are presented as mean ± standard deviation. *P* <0.05 was considered significant.

## Results

**Figure 2:**
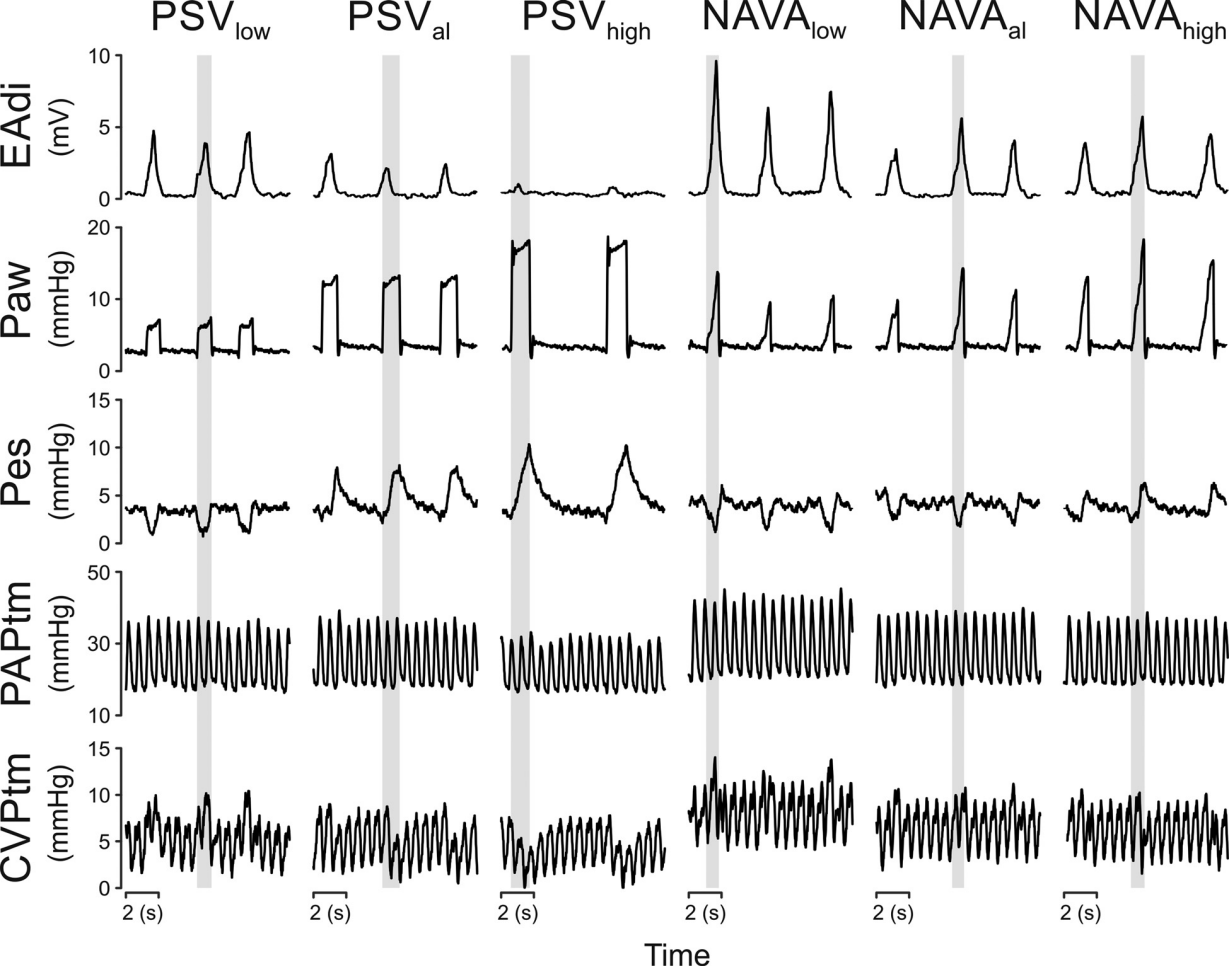
**Breathing pattern and cyclic transmural pressure changes in an individual patient (Patient 8).** Negative deflections in esophageal pressure (Pes) were preserved during inspiration with all neurally adjusted ventilatory assist (NAVA) levels, whereas Pes deflections were negative only with the lowest pressure support ventilation (PSV) level and progressively positive with the adequate PSV (PSVal) and the highest PSV level (PSVhigh). Transmural pressures were not much affected by ventilation with NAVA regardless of the assist level used, whereas the negative cyclic changes during inspiration increased with increasing PSV levels. Vertical grey bars indicate the inspiration (defined by airflow) of the corresponding breath. CVP, central venous pressure; EAdi, electrical activity of the diaphragm; NAVAal, adequate NAVA; NAVAhigh, highest level with constant breathing pattern or a maximum 200% of NAVAal; NAVAlow, 50% of NAVAal; PAP, pulmonary artery pressure; Paw, airway pressure; PSVhigh, 150% of PSVal; PSVlow, 50% of PSVal.

Ten patients (age 66 ± 10 years; five females; Simplified Acute Physiology Score II 41 ± 9) were studied (Table 1). Figure 2 illustrates the respiratory pattern and cyclic intravascular pressure changes for all experimental conditions in a single patient.

**Table 1:** **Characteristics of individual patients**

**Patient number**	**PBW (kg)**	**BMI (kg/m** ^**2**^ **)**	**SAPS II**	**ICU LOS (days)**	**Perioperative LVEF (%)**	**Prestudy PAOP (mmHg)**	**Prestudy cardiac index (l/minute/m** ^**2**^ **)**	**Dobutamine during study (μg/kg/minute)**	**Cardiopulmonary diagnosis**
1	72	26	42	1.8	35	15	1.4	3.1	CABG
2	60	24	34	0.9	31	14	2.6	3.8	CABG
3	57	30	37	1.0	40	12	3.2	0.0	CABG, COPD with cor pulmonale
4	50	31	35	0.9	35	8	1.6	3.4	CABG, aortic valve replacement
5	73	31	54	3.4	35	13	3.6	0.0	Cardiogenic shock following myocardial infarction
6	52	23	40	0.9	30	13	2.2	4.5	CABG, aortic valve replacement, COPD
7	53	34	26	3.7	65	17	2.2	2.9	Aortic valve and arch replacement (type A dissection)
8	73	34	37	3.8	36	16	2.8	0.0	Aortic and mitral valve replacement, COPD
9	64	30	57	6.9	30	11	3.0	3.9	CABG, aortic valve replacement, mitral ring
10	69	24	44	19.8	25	26	3.1	0.0	Cardiogenic shock following myocardial infarction

BMI, body mass index; CABG, coronary artery bypass graft; COPD, chronic obstructive pulmonary disease; LOS, length of stay; LVEF, left ventricular ejection fraction; PAOP, pulmonary artery occlusion pressure; PBW, predicted body weight; SAPS, Simplified Acute Physiology Score.

Five patients with reduced left ventricular ejection fraction and one patient with normal left ventricular ejection fraction (Patient 7) received inotropic drug support (dobutamine, mean dose 2.2 ± 1.9 μg/kg/minute), and four patients with reduced left ventricular ejection fraction did not receive inotropic medication. Biological valves were used for all valve surgery procedures.

NAVAal was identified at 3.1 ± 1.1 cmH_2_O/μV and PSVal at 17 ± 2 cmH_2_O, with a PEEP of 7 ± 2 cmH_2_O (Additional file 5). EAdi was lower with PSV than with NAVA. The tidal volume and transpulmonary pressure increased from NAVAlow to NAVAal and did not increase further at NAVAhigh, whereas both parameters increased with increasing PSV (Table 2 and Additional file 6). Table 2 presents data on the respiratory pattern and the systemic hemodynamic function.

**Table 2:** **Blood gases, respiratory pattern and systemic hemodynamic data**

	**PSVlow**	**PSVal**	**PSVhigh**	**NAVAlow**	**NAVAal**	**NAVAhigh**	***P*** **value**		
							**Level**	**Mode**	**Interaction**
**Blood gases**									
PaO_2_ (mmHg)	94 ± 21	94 ± 21	97 ± 21	94 ± 25	91 ± 25	94 ± 24	0.325	0.495	0.864
PaCO_2_ (mmHg)	38 ± 9	37 ± 8	36 ± 8	39 ± 10	39 ± 11	38 ± 9	<0.001	0.202	0.102
SaO_2_ (%)	97 ± 2	97 ± 1	98 ± 2	97 ± 2	97 ± 2	97 ± 2	0.174	0.309	0.782
pH	7.41 ± 0.1	7.43 ± 0.1	7.45 ± 0.1	7.42 ± 0.1	7.43 ± 0.1	7.43 ± 0.1	<0.001	0.464	0.164
Base excess	0.2 ± 3.6	0.3 ± 3.6	0.1 ± 3.6	0.4 ± 3.5	0.5 ± 3.5	0.5 ± 3.5	0.194	0.244	0.592
**Respiratory pattern**									
Respiratory rate (breaths/minute)	21 ± 5	18 ± 4	13 ± 3	20 ± 5	21 ± 5	20 ± 6	<0.001	0.041	<0.001
Tidal volume (ml/kg PBW)	6.3 ± 1.1	8.2 ± 1.8	11.4 ± 3.0	6.7 ± 1.2	7.0 ± 1.2	7.6 ± 1.2	<0.001	0.018	0.001
Minute ventilation (l/minute)	7.7 ± 2.0	8.6 ± 2.5	8.7 ± 2.1	8.1 ± 2.7	8.6 ± 2.5	8.7 ± 3.0	<0.001	0.619	0.361
Electrical activity of the diaphragm (% EAdi max)	61 ± 16	32 ± 11	17 ± 9	90 ± 33	71 ± 19	29 ± 25	<0.001	0.002	0.3
Mean inspiratory transpulmonary pressure (cmH_2_O)	3 ± 4	5 ± 4	10 ± 4	3 ± 3	3 ± 4	7 ± 5	<0.001	0.158	<0.001
Mean esophageal pressure deflection (cmH_2_O)	−0.7 ± 1.5	2.1 ± 1.9	4 ± 2.3	−1.5 ± 2.4	−1.5 ± 1.7	0 ± 1	0.001	<0.001	<0.001
**Systemic hemodynamic data**									
Pulmonary artery occlusion pressure (mmHg)	16 ± 7	14 ± 4	15 ± 7	15 ± 7	15 ± 5	16 ± 6	0.516	0.424	0.241
Mean systemic arterial pressure (mmHg)	65 ± 8	61 ± 9	65 ± 9	65 ± 9	63 ± 9	63 ± 9	0.065	0.922	0.256
Heart rate (beats/minute)	90 ± 9	90 ± 9	89 ± 9	89 ± 10	90 ± 10	88 ± 10	0.3	0.831	0.567
Cardiac output (l/minute)	4.7 ± 0.9	4.8 ± 0.7	4.8 ± 0.7	5.2 ± 1.2	5.1 ± 1.1	5.1 ± 1.2	0.964	0.082	0.284
Mixed venous oxygen saturation (%)	66 ± 7	64 ± 5	66 ± 9	63 ± 5	65 ± 7	62 ± 5	0.469	0.059	0.211

Data presented as mean ± standard deviation. *P* values from repeated-measures analysis of variance (within-subject factors: ventilation mode, support level). NAVA, neurally adjusted ventilator assist; NAVAal, adequate NAVA level identified by a titration procedure; NAVAhigh, 200% of the adequate NAVA level; NAVAlow, 50% of the adequate NAVA level; PaCO_2_, partial pressure of carbon dioxide; PaO_2_, partial pressure of oxygen; PBW, predicted body weight; PSV, pressure support ventilation; PSVal, adequate level of pressure support ventilation identified on clinical grounds; PSVhigh, 150% of adequate PSV level; PSVlow, 50% of adequate PSV level; SaO_2_, oxygen saturation in arterial blood.

### Transmural pressures

There was no difference between the modes in intravascular or transmural CVP and PAP in expiration. The cyclic changes (inspiratory minus expiratory values) in transmural CVP were small with both NAVA and PSV, and decreased with increasing level of support more prominently with PSV (*P* = 0.03, level*mode interaction). The cyclic changes in transmural PAP were negative for all NAVA levels and PSVlow, and were positive for PSVal and PSVhigh (*P* = 0.026, level*mode interaction; Table 3). The inspiratory deflections in Pes became progressively positive with increasing PSV, while they remained negative with all NAVA levels (*P* < 0.001, level*mode interaction).

**Table 3:** **Mean central venous and mean pulmonary artery pressures for the entire experimental period**

	**PSVlow**	**PSVal**	**PSVhigh**	**NAVAlow**	**NAVAal**	**NAVAhigh**	***P*** **value**		
							**Level**	**Mode**	**Interaction**
**Expiratory values**									
Central venous pressure (mmHg)	10 ± 3	10 ± 3	10 ± 3	11 ± 3	10 ± 3	11 ± 3	0.064	0.091	0.919
Mean pulmonary artery pressure (mmHg)	30 ± 8	27 ± 6	27 ± 6	30 ± 5	29 ± 5	28 ± 5	0.002	0.065	0.208
Transmural central venous pressure (mmHg)	3 ± 4	1 ± 5	2 ± 4	5 ± 4	2 ± 3	3 ± 3	0.06	0.267	0.566
Transmural mean pulmonary artery pressure (mmHg)	22 ± 9	18 ± 8	19 ± 7	24 ± 6	21 ± 5	21 ± 6	<0.001	0.091	0.683
**Cyclic pressure changes**									
Central venous pressure (mmHg)	−0.5 ± 0.4	0.4 ± 0.4	1.0 ± 0.4	−1 ± 0.5	−0.8 ± 0.6	−0.5 ± 0.7	<0.001	<0.001	<0.001
Mean pulmonary artery pressure (mmHg)	−2.2 ± 1.1	0.5 ± 1.4	2.5 ± 1.3	−3.2 ± 0.9	−2.7 ± 1.2	−1.9 ± 1	<0.001	<0.001	<0.001
Transmural central venous pressure (mmHg)	1.3 ± 1.6	0 ± 0.9	−0.9 ± 1.1	1.4 ± 2.4	1.6 ± 1.6	0.1 ± 1.8	0.009	0.08	0.03
Transmural mean pulmonary artery pressure (mmHg)	−0.3 ± 1.8	0.1 ± 0.8	0.5 ± 0.8	−0.8 ± 2.1	−0.4 ± 1.3	−1.2 ± 1.9	0.479	0.078	0.026

Data presented as mean ± standard deviation. Central venous and pulmonary artery pressures (zeroed to atmosphere) and their respective transmural values for the different experimental periods. The transmural values were calculated by subtraction of the esophageal pressure. Cyclic pressure changes are the differences between mean inspiratory and mean expiratory pressures. Data reflect mean values from the analysis of the entire experimental periods. *P* values from repeated-measures analysis of variance (within-subject factors: ventilation mode, support level). NAVA, neurally adjusted ventilator assist; NAVAal, adequate NAVA level identified by a titration procedure; NAVAhigh, 200% of the adequate NAVA level; NAVAlow, 50% of the adequate NAVA level; PSV, pressure support ventilation; PSVal, adequate level of pressure support ventilation identified on clinical grounds; PSVhigh, 150% of adequate PSV level; PSVlow, 50% of adequate PSV level.

**Figure 3:**
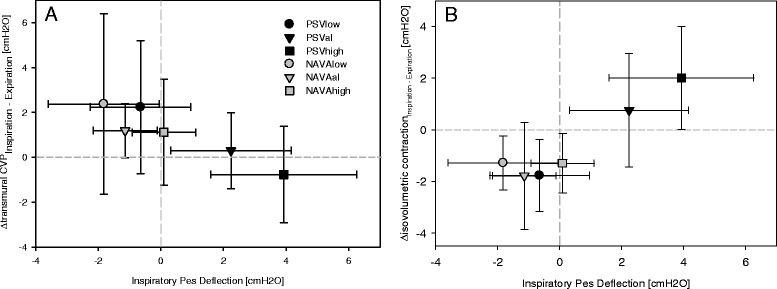
**Loading conditions of the right ventricle in the average breath.** Analysis of the single representative breath cycle. Central venous pressure was measured at the base of the c wave. **(A)** Inspiratory minus expiratory transmural central venous pressure (∆CVP), *P* = 0.015 for level and mode. **(B)** Inspiratory minus expiratory transmural isvolumetric pressure (∆isovolumetric pressure), *P* = 0.003 for support level–ventilation mode interaction. Values presented as mean ± standard deviation. NAVA, neurally adjusted ventilator assist; NAVAal, adequate NAVA level identified by a titration procedure; NAVAhigh, 200% of the adequate NAVA level; NAVAlow, 50% of the adequate NAVA level; Pes, esophageal pressure; PSV, pressure support ventilation; PSVal, adequate level of pressure support ventilation identified on clinical grounds; PSVhigh, 150% of adequate PSV level; PSVlow, 50% of adequate PSV level.

In the analysis of the representative single breath (Figure 3, Table 4), the cyclic changes in transmural CVP decreased with increasing support with both NAVA and PSV (level effect *P* = 0.015). The transmural CVP was higher in inspiration than in expiration at all NAVA levels, whereas increasing PSV resulted in higher CVP during expiration than during inspiration (mode effect *P* = 0.015)

**Table 4:** **Loading conditions of the right ventricle in the average breath**

	**PSVlow**	**PSVal**	**PSVhigh**	**NAVAlow**	**NAVAal**	**NAVAhigh**	***P*** **value**		
							**Level**	**Mode**	**Interaction**
**Transmural central venous pressures at c wave**									
End expiratory (mmHg)	2.6 ± 4	1.3 ± 5.1	2.3 ± 4.1	4.2 ± 4.5	2.4 ± 2.5	3.1 ± 3.4	0.331	0.018	0.817
End inspiratory (mmHg)	4.8 ± 4.5	1.5 ± 4.3	1.5 ± 3.9	6.6 ± 3.0	3.6 ± 3.3	4.2 ± 2.2	0.001	0.1	0.697
Cyclic change (mmHg)	2.2 ± 3	0.3 ± 1.7	−0.8 ± 2.1	2.4 ± 4	1.2 ± 1.2	1.1 ± 2.4	0.015	0.015	0.303
**Isovolumetric pressure generation**									
Inspiratory transmural isovolumetric pressure (mmHg)	11 ± 4.6	12.3 ± 4.4	14 ± 4.6	11.5 ± 4.7	11.1 ± 4.6	10.8 ± 4.2	0.057	0.095	0.005
Expiratory transmural isovolumetric pressure (mmHg)	12.7 ± 4.7	11.5 ± 4.5	12 ± 5.2	12.8 ± 4.1	12.8 ± 3.7	12.1 ± 4.7	0.266	0.257	0.318
Cyclic change in transmural isovolumetric pressure (mmHg)	−1.8 ± 1.4	0.8 ± 2.2	2 ± 2	−1.3 ± 1	−1.8 ± 2.1	−1.3 ± 1.1	0.003	0.009	0.003
**Total pressure generation**									
Inspiratory transmural pressure generation (mmHg)	29.7 ± 10.7	27.2 ± 7.8	29.6 ± 9.4	29.8 ± 9.6	26.9 ± 8.5	26.9 ± 7	0.06	0.38	0.561
Expiratory transmural pressure generation (mmHg)	30.4 ± 10.8	23.5 ± 8.3	26.5 ± 9.4	28.6 ± 10.4	27.9 ± 8	27 ± 6.9	0.231	0.053	0.241
Cyclic change in transmural pressure generation (mmHg)	−0.7 ± 2.3	3.8 ± 8	3.1 ± 1.5	1.2 ± 3.7	−1 ± 4.5	0 ± 2	0.543	0.076	0.033

Data presented as mean ± standard deviation. Data derived from the analysis of the single average breath with measurement of the central venous pressure (CVP) at the base of the c wave. Isovolumetric pressure generation was calculated as the difference between pulmonary artery pressure at valve opening and CVP at the c wave, and the total pressure generation as systolic pulmonary artery pressure minus CVP at the c wave. *P* values from repeated-measures analysis of variance (within-subject factors: ventilation mode, support level). NAVA, neurally adjusted ventilator assist; NAVAal, adequate NAVA level identified by a titration procedure; NAVAhigh, 200% of the adequate NAVA level; NAVAlow, 50% of the adequate NAVA level; PSV, pressure support ventilation; PSVal, adequate level of pressure support ventilation identified on clinical grounds; PSVhigh, 150% of adequate PSV level; PSVlow, 50% of adequate PSV level.

.

### Right ventricular ejection

The transmural inspiratory isovolumetric pressure change increased with increasing pressure support, whereas it remained unchanged with increasing NAVA levels (*P* = 0.005, level*mode interaction; Table 4). Accordingly, the cyclic alterations (inspiratory minus expiratory values) in the isovolumetric pressure change increased with increasing pressure support and remained unchanged with NAVA (*P* = 0.003, level*mode interaction; Figure 3). A similar pattern was observed for the cyclic changes in the total transmural pressure generation of the right ventricle from central venous end diastolic pressure to systolic pulmonary artery pressure (*P* = 0.033, level*mode interaction).

**Figure 4:**
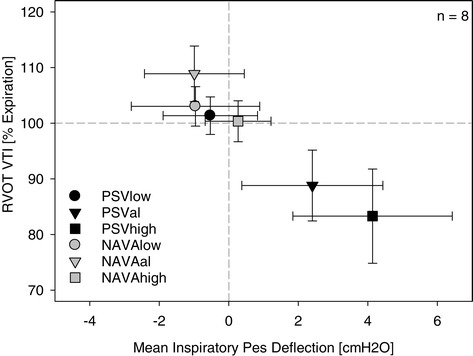
**Right ventricular outflow during the respiratory cycle.** The velocity time integral in the inspiratory right ventricular outflow tract (RVOT VTI) as a percentage of its expiratory value. There is a significant increase in RVOT VTI during inspiration for all NAVA levels compared to PSV (*P* < 0.001, level*mode interaction). Data presented as mean ± standard deviation. NAVA, neurally adjusted ventilator assist; NAVAal, adequate NAVA level identified by a titration procedure; NAVAhigh, 200% of the adequate NAVA level; NAVAlow, 50% of the adequate NAVA level; Pes, esophageal pressure; PSV, pressure support ventilation; PSVal, adequate level of pressure support ventilation identified on clinical grounds; PSVhigh, 150% of adequate PSV level; PSVlow, 50% of adequate PSV level.

Both the ventilation mode and support level modified the right ventricular Doppler flow patterns (Table 5, Figure 4). During inspiration the flow period and the velocity time integral in the right ventricular outflow tract (RVOT VTI, a surrogate of stroke volume) both progressively decreased with increasing PSV level, whereas they remained unchanged with increasing NAVA levels (*P* = 0.028 for flow period and *P* = 0.025 for RVOT VTI, level*mode interaction).

**Table 5:** **Right ventricular echocardiographic data**

**Doppler flow profile in the right ventricular outflow tract (** ***n*** **= 8)**	**PSVlow**	**PSVal**	**PSVhigh**	**NAVAlow**	**NAVAal**	**NAVAhigh**	***P*** **value**		
							**Level**	**Mode**	**Interaction**
**End expiration**									
Acceleration time (milliseconds)	82 ± 14	83 ± 13	80 ± 11	82 ± 16	77 ± 14	89 ± 18	0.484	0.68	0.141
Flow period (milliseconds)	242 ± 25	256 ± 36	256 ± 37	240 ± 23	242 ± 27	243 ± 28	0.072	0.127	0.285
Maximum flow velocity (m/second)	0.8 ± 0.2	0.9 ± 0.3	0.8 ± 0.3	0.7 ± 0.3	0.8 ± 0.2	0.9 ± 0.3	0.047	0.012	0.465
Velocity time integral (cm)	13 ± 2	14 ± 3	14 ± 3	13 ± 2	13 ± 3	14 ± 3	0.009	0.063	0.178
**End inspiration**									
Acceleration time (milliseconds)	86 ± 15	81 ± 15	84 ± 17	81 ± 12	88 ± 20	91 ± 17	0.62	0.259	0.296
Flow period (milliseconds)	250 ± 27	242 ± 25	231 ± 33	250 ± 21	261 ± 29	251 ± 30	0.02	0.067	0.028
Maximum flow velocity (m/second)	0.8 ± 0.2	0.8 ± 0.3	0.7 ± 0.2	0.7 ± 0.3	0.9 ± 0.3	0.8 ± 0.3	0.168	0.154	0.166
Velocity time integral (cm)	13 ± 3	13 ± 3	12 ± 3	13 ± 2	14 ± 3	14 ± 3	0.154	0.046	0.025
**Inspiratory values as percentage of expiratory values**									
cAcceleration time (%)	104 ± 8	98 ± 8	105 ± 10	101 ± 13	115 ± 9	104 ± 11	0.617	0.217	0.012
Flow period (%)	103 ± 2	94 ± 3	91 ± 2	104 ± 2	107 ± 3	103 ± 2	<0.001	<0.001	<0.001
Maximum flow velocity (%)	99 ± 2	92 ± 7	86 ± 8	100 ± 3	106 ± 5	97 ± 4	<0.001	0.003	<0.001
Velocity time integral (%)	101 ± 3	89 ± 6	83 ± 9	103 ± 4	109 ± 5	100 ± 4	<0.001	<0.001	<0.001

Data presented as mean ± standard deviation. Doppler flow profiles in the right ventricular outflow tract could be obtained from eight patients. Absolute values at end expiration did not differ between NAVA and PSV. To describe cyclic changes, the inspiratory values are given as a percentage of the corresponding expiratory value. *P* values from repeated-measures analysis of variance (within-subject factors: ventilation mode, support level). NAVA, neurally adjusted ventilator assist; NAVAal, adequate NAVA level identified by a titration procedure; NAVAhigh, 200% of the adequate NAVA level; NAVAlow, 50% of the adequate NAVA level; PSV, pressure support ventilation; PSVal, adequate level of pressure support ventilation identified on clinical grounds; PSVhigh, 150% of adequate PSV level; PSVlow, 50% of adequate PSV level.

RVOT VTI was equal or higher during inspiration compared with expiration for all NAVA levels. With PSV, RVOT VTI was equal during inspiration and expiration only with PSVlow but it was clearly lower during inspiration compared with expiration with PSVal and PSVhigh (*P* < 0.001, level*mode interaction). The highest RVOT VTI during inspiration compared with expiration was observed with NAVAal (109 ± 5%). The right ventricular stroke volume, as reflected by the RVOT VTI, during inspiration was thus preserved with all NAVA levels whereas it progressively decreased with increasing PSV level.

The left ventricular echocardiography results are summarized in Additional file 7. No relevant differences were observed between the modes.

## Discussion

The main finding of this study was that ventilation with NAVA in patients with impaired cardiac function but stable hemodynamics avoided an inspiratory increase in right ventricular outflow impedance by preserving the cyclic, negative deflections in intrathoracic pressure via feedback control of transpulmonary pressure and tidal volume. The hemodynamic pattern during NAVA closely resembled that of unassisted spontaneous breathing, regardless of the NAVA level used. In contrast, increased inspiratory assist with PSV progressively increased right ventricular outflow impedance, transpulmonary pressure, and tidal volume.

The pattern of decreasing right and increasing left ventricular stroke volume during inspiration is the characteristic pattern of heart–lung interactions during positive pressure ventilation [13-16]. The mechanisms of stroke volume variation during the breathing cycle include changes in venous return and consequent ventricular filling [17-19], and changes in outflow impedance [2,12]. Although these mechanisms have been known for decades, their implications and relevance during NAVA have not been addressed. Specifically, the actual pleural and transmural pressure profiles resulting from clinical application of NAVA will define the cardiac effects of NAVA. These have not been described before. In this respect, our study provides mechanistic insight into the heart–lung interactions of NAVA in a clinically relevant and realistic setting. We used RVOT VTI as a surrogate for right ventricular stroke volume assuming that the pulmonary valve annulus area does not vary with respiration [2]. The progressive reduction in inspiratory RVOT VTI, flow period and maximum flow velocity, and inspiratory increases in right ventricular isovolumetric contraction pressure with increasing PSV indicate increased impedance to right ventricular outflow [12]. In contrast, during all NAVA levels, right ventricular outflow impedance was lower during inspiration than during expiration, as indicated by increased inspiratory RVOT VTI, flow period and maximum flow velocity and reduced right ventricular isovolumetric contraction pressure. Reduced right ventricular afterload during inspiration with NAVA and increased inspiratory afterload with PSV is further supported by negative correlation between inspiratory changes in RVOT VTI and Pes (reflecting pleural pressure; Figure 4) and the decreased RVOT VTI with increased pressures in the pressure/flow diagrams (Additional file 8) [20].

Reduced right ventricular preload due to positive pressure ventilation-associated reduction in venous return [17,19,21] was also likely to contribute to changes in RVOT VTI. Since we did not measure pericardial pressure, we have no direct estimates of right ventricular end-diastolic wall tension. The transmural pressure at the base of the c wave of the CVP tracing, a surrogate of right ventricular end-diastolic wall tension, decreased slightly but significantly with increasing support both in inspiration and expiration without difference between modes. This result should be interpreted with caution, since pleural pressure may not adequately reflect pericardial pressure [22]. Nevertheless, even if changes in preload were involved, the increased right ventricular isovolumetric contraction pressure in the presence of reduced RVOT VTI indicates increased impedance to right ventricular ejection [12] in inspiration with PSV, and the opposite changes indicate reduced impedance to ejection during inspiration with NAVA. The increase in the right ventricle afterload has been assumed to be relevant mainly at high lung volumes [23-25]. Our results clearly indicate the presence of this mechanism also at low tidal volumes, although it did not compromise cardiac performance in our patients with stable hemodynamics to a clinically relevant extent.

Mean inspiratory Pes deflection increased progressively with increasing PSV, whereas the initially slightly negative Pes deflection approached zero with increasing NAVA levels, but did not increase further. This may be related to a progressive downregulation of EAdi with increasing NAVA level, and might limit the effect of NAVA on right ventricular function. Such feedback mechanisms have been previously observed in healthy volunteers and mechanically ventilated patients with acute respiratory failure [6-8,26].

### Study limitations

Several limitations must be addressed. The study was carried out to demonstrate the underlying physiology; due to safety concerns, patients with impaired cardiac function but stable hemodynamic and metabolic conditions were studied for short periods only. Hence, the relevance and consequences of treatment, if any, of these potentially beneficial effects of NAVA should be evaluated further in hemodynamically unstable patients with compromised right ventricular function and in patients with deranged metabolic conditions or with hypercapnia or hypoxemia – clinical conditions where substantial unloading of respiratory muscles with minimum compromise of right ventricular function may be considered.

Most of the patients were studied during the first day after cardiac surgery. Despite being clinically stable, some of these patients could have had relevant intravascular volume changes. In fact, hypovolemia is known to aggravate cyclic intravascular pressure swings [27-29]. Despite potential differences in preload status, the observed hemodynamic response seem consistent within and among individual patients.

There is no standard approach to titrate NAVA. We previously used the same NAVAal titration for several days in ICU patients with critical illness associated polyneuromyopathy (CIPM) without progression to respiratory distress, suggesting that NAVAal provides sufficient respiratory support [8].The average PSV adjusted by the clinicians in charge was based on clinical evaluation of patient comfort, and as a substitute for inspiratory effort, to minimize the cyclic changes in intrathoracic intravascular pressures. The resulting relatively high PSV can be criticized for augmenting the effects of PSV on right ventricular function. However, most patients were likely to have reduced chest wall compliance due to obesity and recent cardiac surgery. The average level of PSV also coincided with the reported average level associated with best patient comfort in difficult to wean patients [30]. PEEP may influence respiratory drive and assist requirements [7], but PEEP was kept constant and should not have influenced the changes within patients. Finally, since PSV and NAVA result in different flow patterns and as flow reversal was used to identify the transition between inspiration and expiration, the investigators could not be blinded to the mode used when analyzing the data.

## Conclusions

In patients with impaired cardiac function, right ventricular performance is less impaired during NAVA titrated to the breathing pattern compared with PSV selected based on clinical criteria. Proposed mechanisms are preservation of cyclic intrathoracic pressure changes characteristic of spontaneous breathing and limitation of right-ventricular outflow impedance during inspiration, regardless of the NAVA level. Thus, NAVA not only prevents lung overdistension and potentially diaphragm disuse, but also the characteristic side effects of positive pressure ventilation on right ventricular function.

## Key messages

- During NAVA, a ventilatory mode that delivers assist in proportion to the neural inspiratory effort, the right-ventricular hemodynamic pattern resembles that of unassisted spontaneous breathing, regardless of the NAVA level used.
- The proposed mechanism includes a lack of increase in right ventricular outflow impedance and a lack of decrease in venous return during lung inflation.
- During NAVA, lung inflation and tidal volume are restricted by neural feedback, while near-zero negative deflections in intrathoracic pressure are preserved.

## Additional files

Additional file 1
**Is an online supplement containing additional details for the** Materials and Methods **section.**
Additional file 2
**Shows the NAVA level titration procedure as described previously** [6-9]. The NAVA level was reduced to a minimum of 0 cmH_2_O/μl resulting in delivery of 2 cmH_2_O (default assist level) when the inspiratory effort exceeded the pneumatic trigger threshold. When sufficient electrical activity of the diaphragm (EAdi, green tracing; middle plot) was detectable, the NAVA level (white tracing; uppermost plot) was manually increased by 0.1 cmH_2_O/μV every 20 seconds. By observing the airway pressure (Paw, yellow tracing; uppermost plot) and tidal volume (Vt, blue tracing; lowermost plot) on the trend screen of the ventilator monitor, NAVAal was determined as the NAVA level early after the transition from an initial steep increase in Paw and Vt (first response) to a less steep increase or even plateau in Paw and Vt (second response). NAVAlow was arbitrarily defined as 50% of NAVAal. NAVAhigh was defined as the highest level resulting in a breathing pattern similar to that observed at NAVAal (that is, before the breathing pattern became unstable and airway pressure started to increase further) [6] or at 200% of NAVAal, whatever occurred first. Additional file 3
**Is a schematic representation of changes in intravascular pressures over the respiratory cycle.** The sample is recorded during ventilation with pressure support (PSV). In order to assess the cyclic change in pressures, the mean expiratory values (represented by the green bar) for central venous, pulmonary artery and systemic arterial pressures were subtracted from their respective mean inspiratory values (represented by the red bar). Thus, a positive result (that is, positive cyclic pressure change) indicates that the pressure was higher during inspiration compared to expiration. Additional file 4
**shows the single-breath analysis.** A single breath (Patient 1, PSVal) was manually selected, based on the mean inspiratory transpulmonary pressure retrieved from the semi-automated analysis. The image thus represents the average breath of this patient in this experimental period. CVP at the base of the c wave (red circles), the opening pressure of the pulmonary valve (blue circles) and the systolic pulmonary artery pressure (green circle) were measured together with their corresponding esophageal pressure (grey circle). The measurements were taken in the cardiac beats closest to end inspiration and end expiration. Additional file 5
**Is a table presenting the individual ventilator settings for all patients studied.** FiO_2_, fraction of inspired oxygen; PEEP, positive end-expiratory pressure; PSV, pressure support ventilation; EAdi, electrical activity of the diaphragm; NAVA, neurally adjusted ventilatory assist; Insp rise, inspiratory rise time. Additional file 6
**Shows the breathing pattern and transpulmonary pressures.** Left: Respiratory cycle and tidal volume for each experimental condition. PBW, predicted body weight [31]. While remaining constant under NAVA, the tidal volume increased (*P* < 0.001 for level*mode interaction) and the duty cycle (the inspiratory proportion total cycle time) and respiratory rate decreased (*P* < 0.005 and *P* < 0.001 respectively, level*mode interaction) with increasing the PSV level. NAVA, neurally adjusted ventilatory assist; PSV, pressure support ventilation; respective support levels, low, al (adequate level), and high. Values presented as mean ± standard deviation. Right: Transpulmonary pressure (Ptp) isobars (dotted ascending lines). Transpulmonary pressure increased from NAVAlow to NAVAal but remained stable when further increasing the assist to NAVAhigh. With PSV the transpulmonary pressure steadily increased from PSVlow to PSVhigh. Mean inspiratory airway pressure (including PEEP) and Ptp were lower during NAVA compared with the corresponding PSV levels (*P* < 0.05 for all, level*mode interaction). Mean inspiratory esophageal pressure deflection was negative for all NAVA levels, whereas it was negative only for PSVlow and positive for PSVal and PSVhigh (*P* < 0.001 for all comparisons). Additional file 7
**Is a table presenting Doppler flow profiles in the left ventricular outflow tract could be obtained from seven patients.** Absolute values at end expiration did not differ between NAVA and PSV. To describe cyclic changes, the inspiratory values are given as a percentage of the corresponding expiratory value. Values are mean ± standard deviation. **P* values from repeated-measures analysis of variance (ANOVA; within-subject factors: ventilation mode, support level). Additional file 8
**Shows the pressure/flow relationships.** To support the concept of increasing inspiratory impedance to flow during PSV, we have constructed figures plotting the cyclic changes (inspiratory minus expiratory values) for absolute mean pulmonary artery pressure (referenced to atmosphere **(A)**) and for transmural mean pulmonary artery pressure (referenced to esophageal pressure **(B)**) against cyclic changes (inspiratory minus expiratory value) in RVOT VTI, assuming that the RVOT VTI is an adequate surrogate of right ventricular (RV) stroke volume [2]. The common starting point in the center represents the expiration, the movement along the vector the change in inspiration. The simultaneous increase in stroke volume and reduction of transmural pulmonary artery pressure during inspiration and increase in RV stroke volume (lower right quadrant) is consistent with reduced RV afterload, whereas increasing PSV leads to reduction of stroke volume and unchanged or increased pulmonary artery pressure during inspiration, which is consistent with increased RV afterload [20]. This analysis assumes similar changes in left atrial pressure from inspiration to expiration [32,33]. Values are means.

## Abbreviations

CVP: central venous pressure
EAdi: electrical activity of the diaphragm
NAVA: neurally adjusted ventilator assist
NAVAal: adequate neurally adjusted ventilator assist level identified by a titration procedure
NAVAhigh: 200% of the adequate neurally adjusted ventilator assist level
NAVAlow: 50% of the adequate neurally adjusted ventilator assist level
PAP: pulmonary artery pressure
PEEP: positive end-expiratory pressure
Pes: esophageal pressure
PSV: pressure support ventilation
PSVal: adequate level of pressure support ventilation identified on clinical grounds
PSVhigh: 150% of adequate pressure support level
PSVlow: 50% of adequate pressure support level
RVOT VTI: velocity time integral in right ventricular outflow tract
