# Supplementary material for: Heart–lung interactions during neurally adjusted ventilatory assist
Source: Crit Care. 2014 Sep 12;18(5):499. doi: 10.1186/s13054-014-0499-8 (PMC4189198; doi:10.1186/s13054-014-0499-8)
Supplement: Additional file 1 — Is an online supplement containing additional details for the Materials and Methods section. [file 13054_2014_499_MOESM1_ESM.pdf]

Heart-Lung Interactions during Neurally Adjusted Ventilatory Assist

Clinical Trial.gov Identifier: NCT00647361

David Berger, Stefan Bloechlinger , Jukka Takala , Christer Sinderby , Lukas Brander

Online Supplemental Material

Additional Method Section

Exclusion criteria

Exclusion criteria were lack of informed consent, pregnancy or breastfeeding, any contraindication to insert a nasogastric tube, diaphragm injury, acute or chronic central nervous system disorder, history of cardiac or lung transplantation, any mechanical cardiac assist, need for neuromuscular blocking agents, any contraindication to reduce or stop sedation, concurrent participation in another interventional clinical trial, need for cardiac pacing other than AAI mode, atrial fibrillation, poor image quality in transthoracic echocardiography.

Safety measures

The study had to be stopped in case of arterial blood desaturation to < 90% (or to < 85 % in patients with a history of chronic obstructive pulmonary disease) unresponsive to an increase of the inspiratory fraction of oxygen to a maximum of 0.8, or in case of hemodynamic instability with a decrease in mean arterial blood pressure > 30% of baseline (=average of the last 30 minutes before starting the study) or to < 55mmHg unresponsive to therapeutic interventions as per written prescriptions by the attending physician.

Installations

As part of routine care all patients were monitored (S/5 Critical Care Monitor™, Datex-Ohmeda, Helsinki, Finland) with an arterial line and a pulmonary artery catheter with continuous measurement of central venous pressure, pulmonary arterial pressure, cardiac output, and mixed

venous oxygenation (CCOmbo, Edwards Lifesciences, California). Esophageal balloon catheters (SmartCath esophageal Catheter, Viasys Healthcare, CareFusion, California) and nasogastric tubes for EAdi measurements (Maquet. Solna, Sweden) were inserted using the occlusion test for the esophageal balloon(1, 2) and a dedicated software provided by the ventilator manufacturer for the EAdi catheter(3) in order to verify correct positioning.

Study Protocol

Titration of an adequate NAVA level to breathing pattern

NAVA principles, catheter positioning and the NAVA level titration procedure have been previously described (3-9).

For identification of an adequate NAVA level (NAVAal) the gain factor was first reduced to 0cmH_2O/μV (i.e. no ventilatory assist was delivered except for a minimal default assist of 2cmH2O when the patient's inspiratory effort exceeded the pneumatic inspiratory trigger threshold). After electrical activity of the diaphragm (EAdi) had stabilized, the NAVA level (i.e. the gain factor) was manually increased in increments of 0.1cmH_2O/μV every 20 seconds. NAVAal was visually identified on trend graphs displayed on the ventilator screen early after the transition from an initial steep increase in airway pressure (Paw) and tidal volume (Vt) (1st response) to a less steep increase (2nd response) (see additional file 2).

Measurements

Ventilatory and hemodynamic parameters

Ventilator pressure (Pvent), airflow and EAdi were continuously recorded using a dedicated software provided by the ventilator manufacturer (NAVA tracker, Maquet, Solna, Sweden). Airway pressure proximal to the Y-piece (Paw) and esophageal pressure (Pes) were measured using a custom made acquisition system (MPX2050DP, Freescale Semiconductor Inc., Arizona, USA) (10) and recorded simultaneously with arterial, central venous and pulmonary artery pressures by an acquisition software (Neurovent Research Inc, Toronto, Canada), as published previously (4, 6, 7). Sampling frequency for all these recordings was 62.5 Hz. Cardiac output and central venous oxygen saturation (Vigilance II, Edwards Lifesciences, Nyon, Switzerland) were recorded by a patient data management system as two minute median values (Clinicare, GE Healthcare, Finland).

Transthoracic Echocardiography

Transthoracic echocardiography (Vivid 7, GE Medical Systems CH, Glattbrugg, Switzerland) was performed by an experienced cardiologist (author SB). The electrocardiogramm and the airway pressure were continuously displayed and recorded for timing the analyses. Parasternal short-axis and apical views were acquired during every experimental period, including the following recordings: Pulsed wave Doppler analysis was performed at the level of the pulmonary valve, in the left ventricular outflow tract and at the tips of the mitral leaflets. Continuous wave Doppler images were recorded from the aortic and tricuspid valve, and tissue Doppler imaging from the septal and

lateral mitral annuli as well as the lateral tricuspid annulus. A parasternal long-axis and an apical 2-chamber view were acquired during PSVal and NAVAal. Images and loops were stored in raw data format for offline analysis using dedicated software (EchoPac, GE Medical Systems CH, Glattbrugg, Switzerland).

Flow across the pulmonary valve was assessed from stored pulsed wave Doppler images on a breath-to-breath basis. In- and expiration were tracked by the airway pressure curve visualized on the Doppler screen. For three end-inspiratory and three end-expiratory cardiac beats (i.e. three single breaths) the velocity time integral, flow period, acceleration time, maximum velocity and the preceding R-R interval in the electrocardiogram were measured and mean end-expiratory and end-inspiratory values were calculated for each patient (11). The same parameters were assessed from stored left ventricular outflow tract Pulsed Wave Doppler images.

Diastolic function was examined and graded according to standard criteria for every respiratory period (12). Right ventricular function was assessed for every respiratory period using tissue velocity of the lateral tricuspid annulus (S') and TAPSE (13, 14). Left ventricular mass was calculated from a loop during PSVal ventilation as recommended by the American Society of Echocardiography (15, 16). Left ventricular ejection fraction was calculated using the biplane Simpson method in PSVal and NAVAal (16). In case the biplane Simpson method was not feasible, a Teichholz measurement combined with a visual estimate was used instead. Valve function and pulmonary artery pressure were assessed according to the American Society of Echocardiography Guidelines (17, 18).

Data analysis

Breathing pattern

Breath-by-breath analysis was performed using custom made software (Neurovent Research Inc, Toronto, Canada). Coughing and artefacts were identified visually and excluded from analyses. For each experimental period, beginning and end of inspiratory airflow were marked off-line using trigger-on and cycling-off thresholds as set during the study. In- and expiration were defined by airflow reversal. We have used similar approaches in our previous studies (4, 6, 7). The mean inspiratory esophageal pressure (Pes) deflection was calculated for the duration of inspiratory airflow and referenced to the Pes immediately before start of inspiratory airflow. For each parameter, the mean value of all analysed breaths was calculated. Transmural vascular pressures were obtained by subtraction of the esophageal pressure from the respective vascular pressure. Conversion of cmH2O to mmHg was done with a factor of 0.735 mmHg/cmHO.

References online supplement

1. Baydur A, Behrakis PK, Zin WA, Jaeger M, Milic-Emili J. A simple method for assessing the validity of the esophageal balloon technique. *The American review of respiratory disease* 1982;126:788-791.

2. Baydur A, Cha EJ, Sassoon CS. Validation of esophageal balloon technique at different lung volumes and postures. *Journal of applied physiology: respiratory, environmental and exercise physiology* 1987;62:315-321.

3. Barwing J, Ambold M, Linden N, Quintel M, Moerer O. Evaluation of the catheter positioning for neurally adjusted ventilatory assist. *Intensive care medicine* 2009;35:1809-1814.

4. Brander L, Leong-Poi H, Beck J, Brunet F, Hutchison SJ, Slutsky AS, Sinderby C. Titration and implementation of neurally adjusted ventilatory assist in critically ill patients. *Chest* 2009;135:695-703.

5. Lecomte F, Brander L, Jalde F, Beck J, Qui H, Elie C, Slutsky AS, Brunet F, Sinderby C. Physiological response to increasing levels of neurally adjusted ventilatory assist (nava). *Respiratory physiology & neurobiology* 2009;166:117-124.

6. Passath C, Takala J, Tuchscherer D, Jakob SM, Sinderby C, Brander L. Physiologic response to changing positive end-expiratory pressure during neurally adjusted ventilatory assist in sedated, critically ill adults. *Chest* 2010;138:578-587.

7. Tuchscherer D, Z'Graggen W J, Passath C, Takala J, Sinderby C, Brander L. Neurally adjusted ventilatory assist in patients with critical illness-associated polyneuromyopathy. *Intensive care medicine* 2011;37:1951-1961.

8. Ververidis D, Van Gils M, Passath C, Takala J, Brander L. Identification of adequate neurally adjusted ventilatory assist (nava) during systematic increases in the nava level. *IEEE transactions on bio-medical engineering* 2011;58:2598-2606.

9. Sinderby C, Navalesi P, Beck J, Skrobik Y, Comtois N, Friberg S, Gottfried SB, Lindstrom L. Neural control of mechanical ventilation in respiratory failure. *Nature medicine* 1999;5:1433-1436.

10. 50 kpa on-chip temperature compensated and calibrated silicon pressure sensors. Freescale Semiconductor; 2008.

11. Vieillard-Baron A, Chergui K, Augarde R, Prin S, Page B, Beauchet A, Jardin F. Cyclic changes in arterial pulse during respiratory support revisited by doppler echocardiography. *Am J Respir Crit Care Med* 2003;168:671-676.

12. Nagueh SF, Appleton CP, Gillebert TC, Marino PN, Oh JK, Smiseth OA, Waggoner AD, Flachskampf FA, Pellikka PA, Evangelista A. Recommendations for the evaluation of left ventricular diastolic function by echocardiography. *Journal of the American Society of Echocardiography : official publication of the American Society of Echocardiography* 2009;22:107-133.

13. Kaul S, Tei C, Hopkins JM, Shah PM. Assessment of right ventricular function using two-dimensional echocardiography. *Am Heart J* 1984;107:526-531.

14. Meluzin J, Spinarova L, Bakala J, Toman J, Krejci J, Hude P, Kara T, Soucek M. Pulsed doppler tissue imaging of the velocity of tricuspid annular systolic motion; a new, rapid, and non-invasive method of evaluating right ventricular systolic function. *European heart journal* 2001;22:340-348.

15. Devereux RB, Alonso DR, Lutas EM, Gottlieb GJ, Campo E, Sachs I, Reichek N. Echocardiographic assessment of left ventricular hypertrophy: Comparison to necropsy findings. *The American journal of cardiology* 1986;57:450-458.

16. Lang RM, Bierig M, Devereux RB, Flachskampf FA, Foster E, Pellikka PA, Picard MH, Roman MJ, Seward J, Shanewise JS, Solomon SD, Spencer KT, Sutton MS, Stewart WJ. Recommendations for chamber quantification: A report from the american society of echocardiography's guidelines and standards committee and the chamber quantification writing group, developed in conjunction with the european association of echocardiography, a branch of the

european society of cardiology. *Journal of the American Society of Echocardiography : official publication of the American Society of Echocardiography* 2005;18:1440-1463.

17. Zoghbi WA, Enriquez-Sarano M, Foster E, Grayburn PA, Kraft CD, Levine RA, Nihoyannopoulos P, Otto CM, Quinones MA, Rakowski H, Stewart WJ, Waggoner A, Weissman NJ. Recommendations for evaluation of the severity of native valvular regurgitation with two-dimensional and doppler echocardiography. *Journal of the American Society of Echocardiography : official publication of the American Society of Echocardiography* 2003;16:777-802.

18. Baumgartner H, Hung J, Bermejo J, Chambers JB, Evangelista A, Griffin BP, Iung B, Otto CM, Pellikka PA, Quinones M. Echocardiographic assessment of valve stenosis: Eae/ase recommendations for clinical practice. *Journal of the American Society of Echocardiography : official publication of the American Society of Echocardiography* 2009;22:1-23; quiz 101-102.
