# Supplementary figures and images for: Heart–lung interactions during neurally adjusted ventilatory assist
Source: Crit Care. 2014 Sep 12;18(5):499. doi: 10.1186/s13054-014-0499-8 (PMC4189198; doi:10.1186/s13054-014-0499-8)

## Slide 1
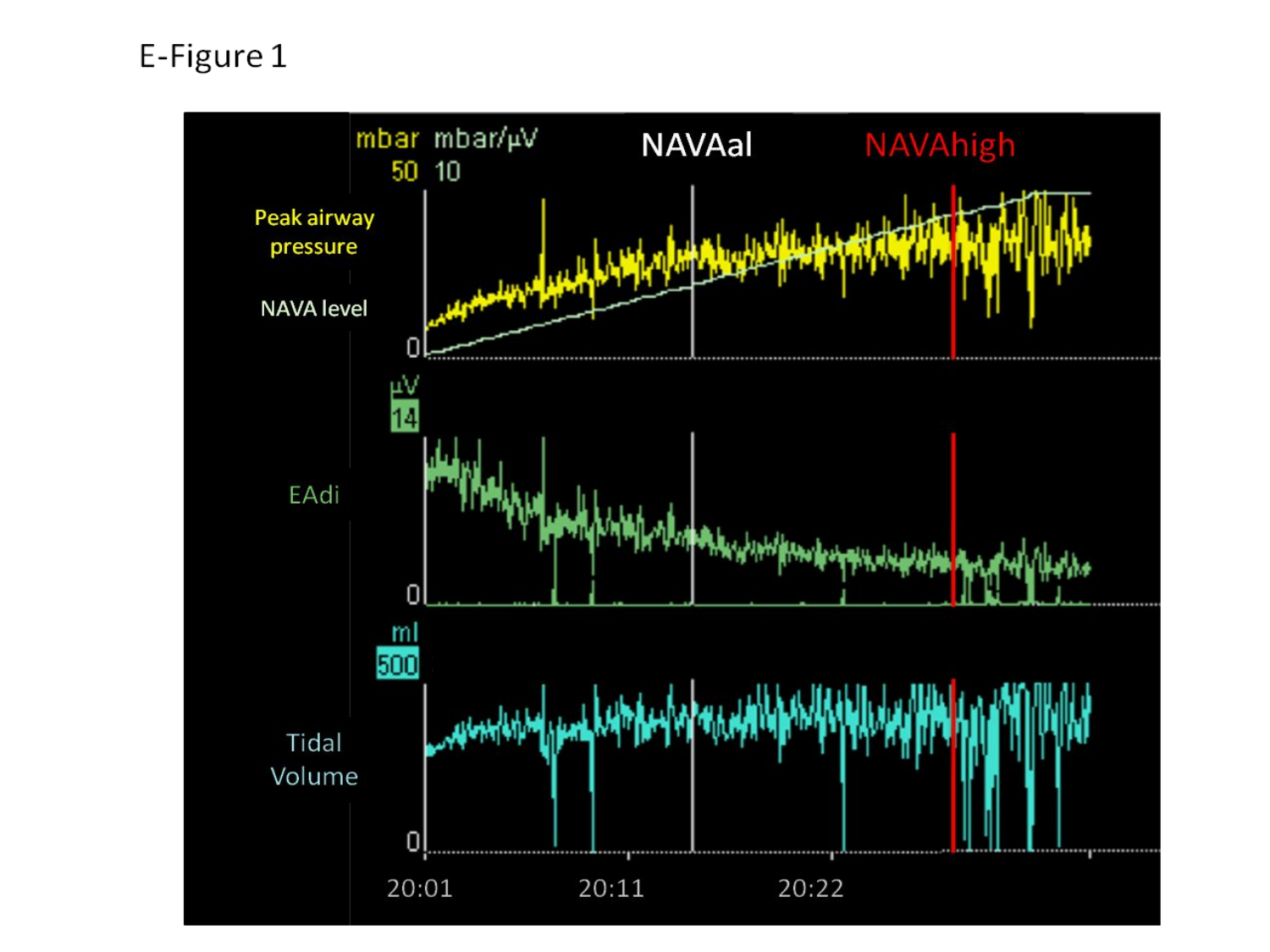

Supplement: Additional file 2 — Shows the NAVA level titration procedure as described previously [6-9]. The NAVA level was reduced to a minimum of 0 cmH2O/μl resulting in delivery of 2 cmH2O (default assist level) when the inspiratory effort exceeded the pneumatic trigger threshold. When sufficient electrical activity of the diaphragm (EAdi, green tracing; middle plot) was detectable, the NAVA level (white tracing; uppermost plot) was manually increased by 0.1 cmH2O/μV every 20 seconds. By observing the airway pressure (Paw, yellow tracing; uppermost plot) and tidal volume (Vt, blue tracing; lowermost plot) on the trend screen of the ventilator monitor, NAVAal was determined as the NAVA level early after the transition from an initial steep increase in Paw and Vt (first response) to a less steep increase or even plateau in Paw and Vt (second response). NAVAlow was arbitrarily defined as 50% of NAVAal. NAVAhigh was defined as the highest level resulting in a breathing pattern similar to that observed at NAVAal (that is, before the breathing pattern became unstable and airway pressure started to increase further) [6] or at 200% of NAVAal, whatever occurred first. [file 13054_2014_499_MOESM2_ESM.pptx]

## Slide 1
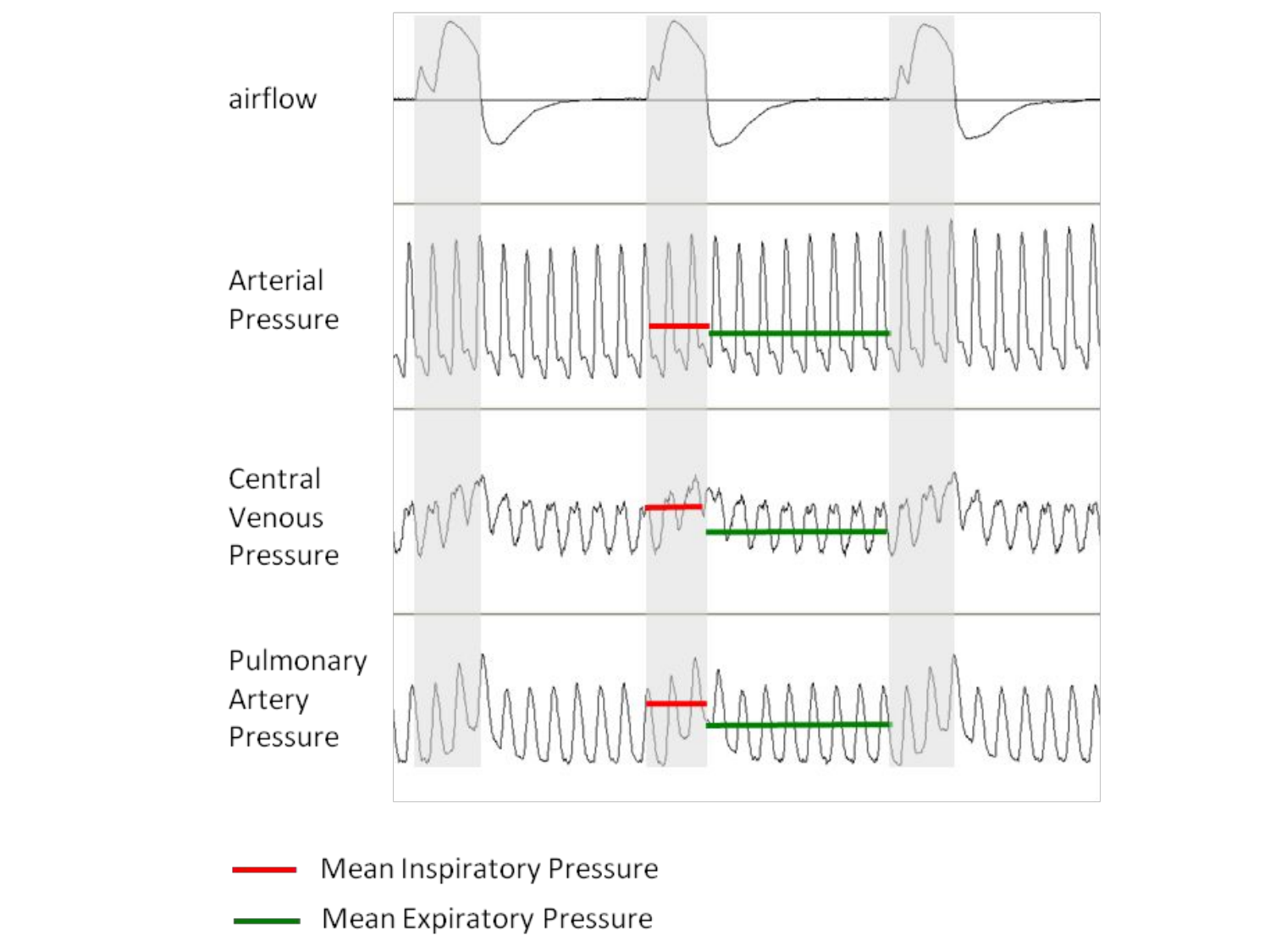

Supplement: Additional file 3 — Is a schematic representation of changes in intravascular pressures over the respiratory cycle. The sample is recorded during ventilation with pressure support (PSV). In order to assess the cyclic change in pressures, the mean expiratory values (represented by the green bar) for central venous, pulmonary artery and systemic arterial pressures were subtracted from their respective mean inspiratory values (represented by the red bar). Thus, a positive result (that is, positive cyclic pressure change) indicates that the pressure was higher during inspiration compared to expiration. [file 13054_2014_499_MOESM3_ESM.pptx]

## Slide 1
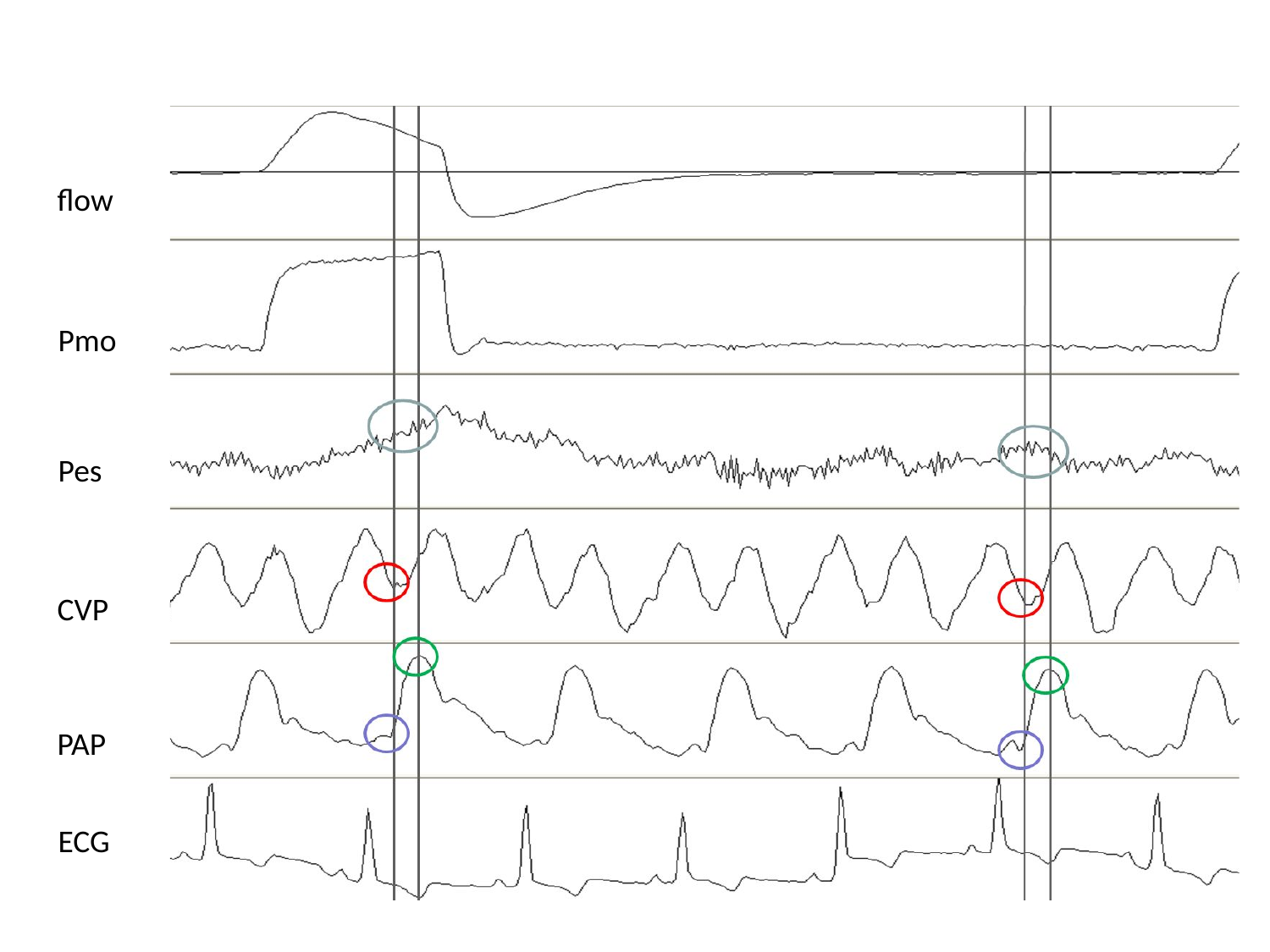

flow
Pmo
Pes
CVP
PAP
ECG

Supplement: Additional file 4 — shows the single-breath analysis. A single breath (Patient 1, PSVal) was manually selected, based on the mean inspiratory transpulmonary pressure retrieved from the semi-automated analysis. The image thus represents the average breath of this patient in this experimental period. CVP at the base of the c wave (red circles), the opening pressure of the pulmonary valve (blue circles) and the systolic pulmonary artery pressure (green circle) were measured together with their corresponding esophageal pressure (grey circle). The measurements were taken in the cardiac beats closest to end inspiration and end expiration. [file 13054_2014_499_MOESM4_ESM.pptx]

## Slide 1
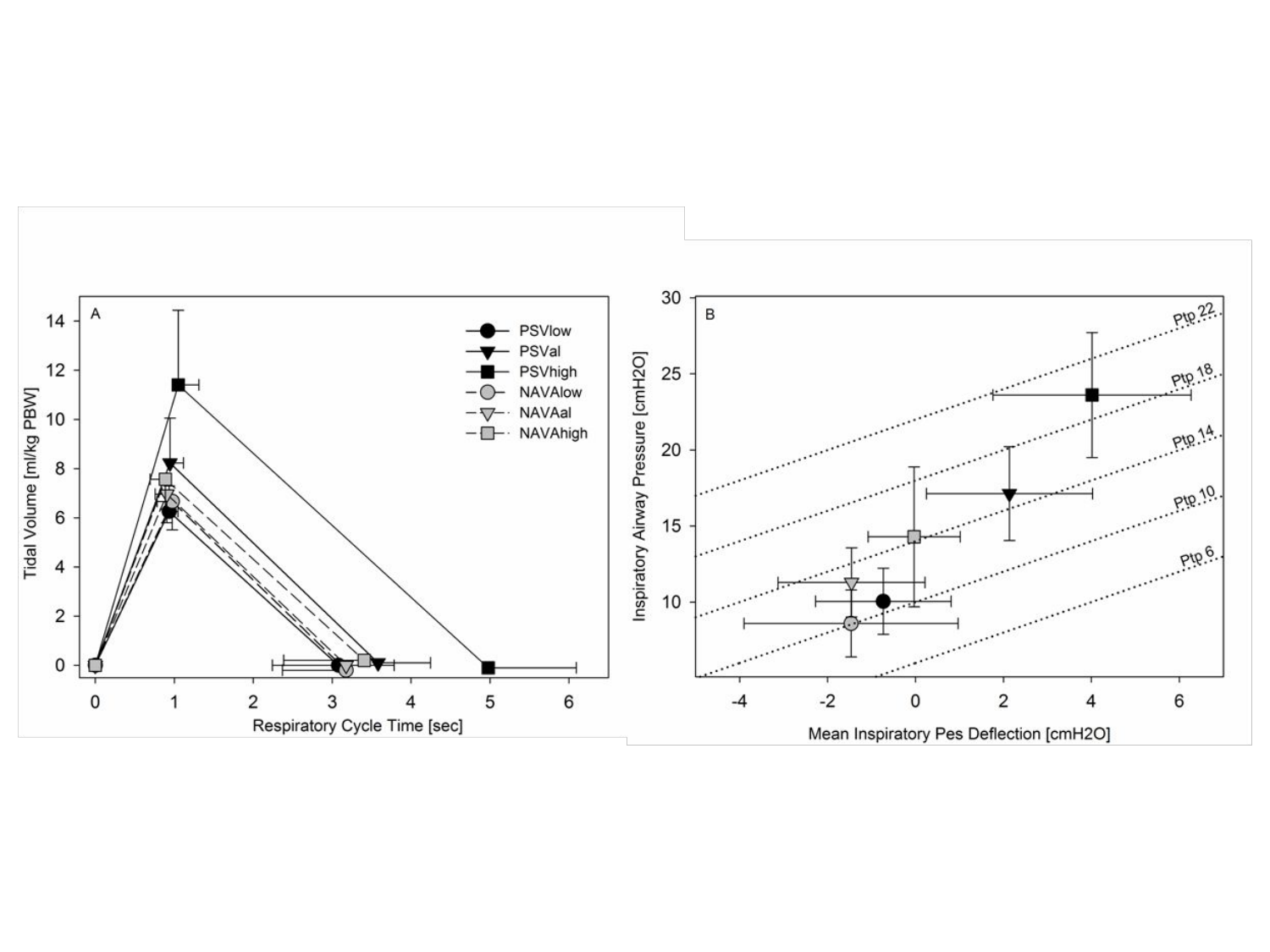

Supplement: Additional file 6 — Shows the breathing pattern and transpulmonary pressures. Left: Respiratory cycle and tidal volume for each experimental condition. PBW, predicted body weight [31]. While remaining constant under NAVA, the tidal volume increased (P < 0.001 for level*mode interaction) and the duty cycle (the inspiratory proportion total cycle time) and respiratory rate decreased (P < 0.005 and P < 0.001 respectively, level*mode interaction) with increasing the PSV level. NAVA, neurally adjusted ventilatory assist; PSV, pressure support ventilation; respective support levels, low, al (adequate level), and high. Values presented as mean ± standard deviation. Right: Transpulmonary pressure (Ptp) isobars (dotted ascending lines). Transpulmonary pressure increased from NAVAlow to NAVAal but remained stable when further increasing the assist to NAVAhigh. With PSV the transpulmonary pressure steadily increased from PSVlow to PSVhigh. Mean inspiratory airway pressure (including PEEP) and Ptp were lower during NAVA compared with the corresponding PSV levels (P < 0.05 for all, level*mode interaction). Mean inspiratory esophageal pressure deflection was negative for all NAVA levels, whereas it was negative only for PSVlow and positive for PSVal and PSVhigh (P < 0.001 for all comparisons). [file 13054_2014_499_MOESM6_ESM.pptx]

## Slide 1
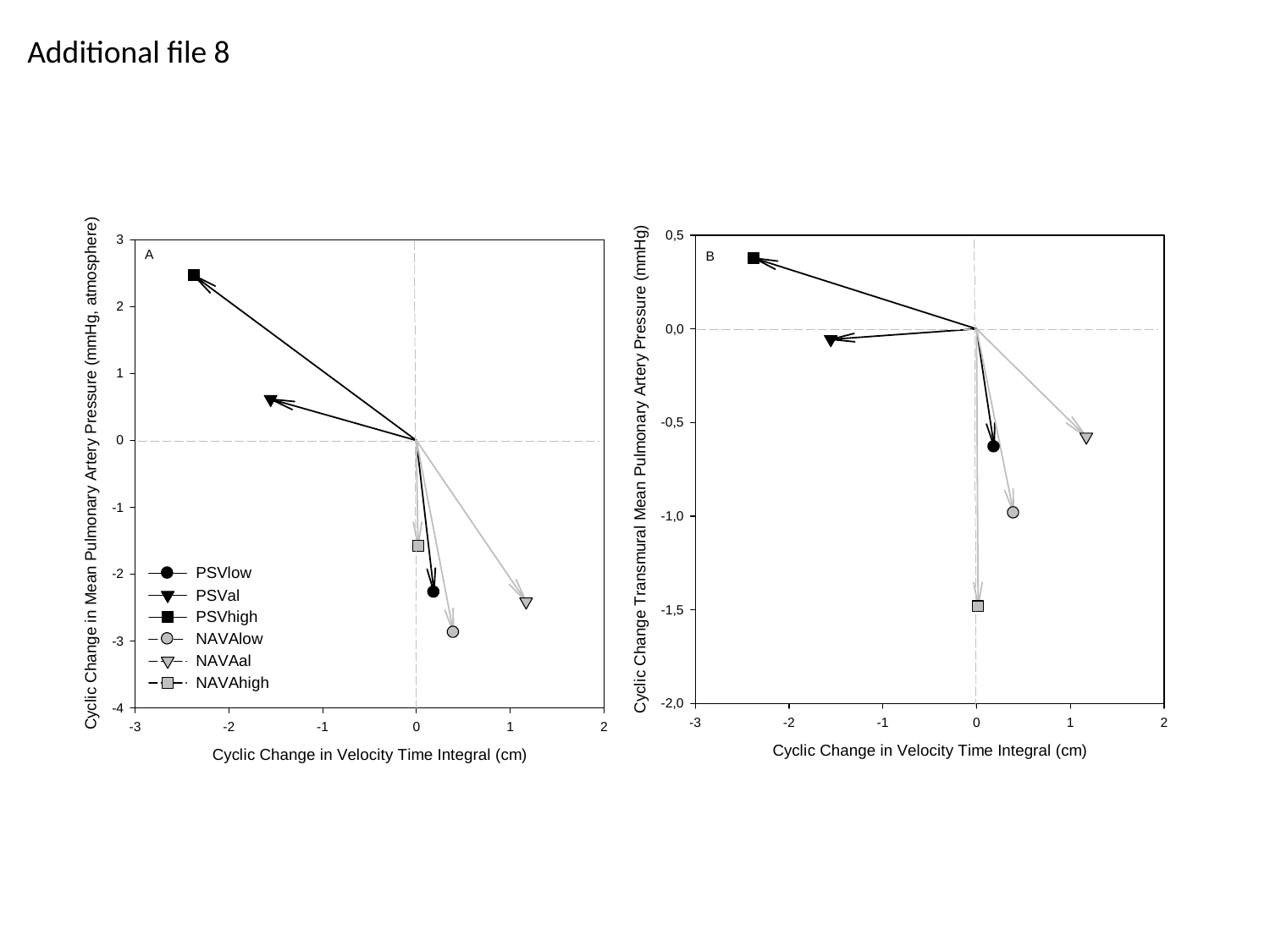

Additional file 8

Supplement: Additional file 8 — Shows the pressure/flow relationships. To support the concept of increasing inspiratory impedance to flow during PSV, we have constructed figures plotting the cyclic changes (inspiratory minus expiratory values) for absolute mean pulmonary artery pressure (referenced to atmosphere (A)) and for transmural mean pulmonary artery pressure (referenced to esophageal pressure (B)) against cyclic changes (inspiratory minus expiratory value) in RVOT VTI, assuming that the RVOT VTI is an adequate surrogate of right ventricular (RV) stroke volume [2]. The common starting point in the center represents the expiration, the movement along the vector the change in inspiration. The simultaneous increase in stroke volume and reduction of transmural pulmonary artery pressure during inspiration and increase in RV stroke volume (lower right quadrant) is consistent with reduced RV afterload, whereas increasing PSV leads to reduction of stroke volume and unchanged or increased pulmonary artery pressure during inspiration, which is consistent with increased RV afterload [20]. This analysis assumes similar changes in left atrial pressure from inspiration to expiration [32,33]. Values are means. [file 13054_2014_499_MOESM8_ESM.pptx]
