# Supplementary material for: Heart–lung interactions during neurally adjusted ventilatory assist
Source: Crit Care. 2014 Sep 12;18(5):499. doi: 10.1186/s13054-014-0499-8 (PMC4189198; doi:10.1186/s13054-014-0499-8)
Supplement: Additional file 5 — Is a table presenting the individual ventilator settings for all patients studied. FiO2, fraction of inspired oxygen; PEEP, positive end-expiratory pressure; PSV, pressure support ventilation; EAdi, electrical activity of the diaphragm; NAVA, neurally adjusted ventilatory assist; Insp rise, inspiratory rise time. [file 13054_2014_499_MOESM5_ESM.pdf]

Additional Table 1: Individual ventilator settings

			Pat 1	Pat 2	Pat 3	Pat 4	Pat 5	Pat 6	Pat 7	Pat 8	Pat 9	Pat 10	Mean±SD
	FiO2	%	40	40	35	40	45	40	60	45	50	50	45±7
	PEEP	cmH2O	5	5	5	5	8	5	10	5	10	8	7±2
	PSV	cmH2O	7	10	7	8	9	7	9	8	9	9	8±1
**PSVlow**	mean Eadi	uvol	1.4	0.6	4.0	2.9	10.5	3.8	1.0	3.7	4.5	4.4	4±3
	Flowtrigger	l/min	5	5	5	5	5	2	6	5	5	7	5±1
	Insp rise	sec	0.0	0.0	0.0	0.0	0.0	0.0	0.0	0.0	0.0	0.0	0.0±0
	cycling off	% max flow	30	30	30	30	25	30	20	30	30	10	27±7
	FiO2	%	40	40	35	40	45	40	60	45	50	50	45±7
	PEEP	cmH2O	5	5	5	5	8	5	10	5	10	8	7±2
	PSV	cmH2O	14	20	14	16	18	14	18	16	18	18	17±2
**PSVal**	mean Eadi	uvol	0.5	0.4	2.9	1.2	5.8	2.3	0.4	1.5	2.2	2.3	2±2
	Flowtrigger	l/min	5	5	5	5	5	2	6	5	5	7	5±1
	Insp rise	sec	0.0	0.0	0.0	0.0	0.0	0.0	0.0	0.0	0.0	0.0	0.0±0
	cycling off	% max flow	30	30	30	30	25	30	20	30	30	10	27±7
	FiO2	%	40	40	35	40	45	40	60	45	50	50	45±7
	PEEP	cmH2O	5	5	5	5	8	5	10	5	10	8	7±2
	PSV	cmH2O	21	30	21	24	27	21	27	24	27	24	25±3
**PSVhigh**	mean Eadi	uvol	0.5	0.3	1.7	1.1	0.7	0.7	0.4	0.7	0.6	1.2	1±0
	Flowtrigger	l/min	5	5	5	5	5	2	6	5	5	7	5±1
	Insp rise	sec	0.0	0.0	0.0	0.0	0.0	0.0	0.0	0.0	0.0	0.0	0.0±0
	cycling off	% max flow	30	30	30	30	25	30	20	30	30	10	27±7
	FiO2	%	40	40	35	40	45	40	60	45	50	50	45±7
	PEEP	cmH2O	5	5	5	5	8	5	10	5	10	8	7±2
	NAVA level	cmH2O/uvol	1.5	1.1	2.1	2.6	1.5	1.2	1.8	1.8	0.9	1	1.6±0.5
**NAVAlow**	mean Eadi	uvol	1.9	1.8	3.6	3.5	8.5	3.5	3.5	5.0	7.7	6.1	5±2
	Flowtrigger	l/min	5	5	5	5	5	5	7	5	5	5	5±1
	Edi Trigger	uvol	0.5	0.5	0.5	0.5	0.5	0.5	0.3	0.3	0.5	0.5	0±0
	cycling off	% max Eadi	70	70	70	70	70	70	70	70	70	70	27±7
	FiO2	%	40	40	35	40	45	40	60	45	50	50	45±7
	PEEP	cmH2O	5	5	5	5	8	5	10	5	10	8	7±2
	NAVA level	cmH2O/uvol	3	2.1	4.3	5.2	3	2.4	3.6	3.6	1.7	1.9	3.1±1.1
**NAVAal**	mean Eadi	uvol	1.8	1.6	3.2	2.4	6.0	3.9	2.1	4.0	5.6	5.2	4±2
	Flowtrigger	l/min	5	5	5	5	5	5	7	5	5	5	5±1
	Edi Trigger	uvol	0.5	0.5	0.5	0.5	0.5	0.5	0.3	0.3	0.5	0.5	0±0
	cycling off	% max Eadi	70	70	70	70	70	70	70	70	70	70	27±7
	FiO2	%	40	40	35	40	45	40	60	45	50	50	45±7
	PEEP	cmH2O	5	5	5	5	8	5	10	5	10	8	7±2
	NAVA level	cmH2O/uvol	6	3.6	7.3	8.6	12	4.8	7.2	4.8	3.4	2.4	6.0±2.9
**NAVAhigh**	mean Eadi	uvol	1.5	1.0	2.3	2.4	2.4	2.8	1.6	3.6	3.8	8.5	3±2
	Flowtrigger	l/min	5	5	5	5	5	5	7	5	5	5	5±1
	Edi Trigger	uvol	0.5	0.5	0.5	0.5	0.5	0.5	0.3	0.3	0.5	0.5	0±0
	cycling off	% max Eadi	70	70	70	70	70	70	70	70	70	70	27±7

Additional Table 1

This table contains the individual ventilator settings for all patients studied.

Definition of abbreviations : FiO2 = fraction of inspired oxygen, PEEP = positive end-expiratory pressure, PSV = pressure support ventilation, EAdi = electrical activity of the diaphragm, NAVA = neurally adjusted ventilatory assist, Insp rise = inspiratory rise time.
