# Supplementary material for: Heart–lung interactions during neurally adjusted ventilatory assist
Source: Crit Care. 2014 Sep 12;18(5):499. doi: 10.1186/s13054-014-0499-8 (PMC4189198; doi:10.1186/s13054-014-0499-8)
Supplement: Additional file 7 — Is a table presenting Doppler flow profiles in the left ventricular outflow tract could be obtained from seven patients. Absolute values at end expiration did not differ between NAVA and PSV. To describe cyclic changes, the inspiratory values are given as a percentage of the corresponding expiratory value. Values are mean ± standard deviation. *P values from repeated-measures analysis of variance (ANOVA; within-subject factors: ventilation mode, support level). [file 13054_2014_499_MOESM7_ESM.pdf]

Additional Table 2. Left Ventricular Echocardiography Data

	PSVlow	PSVal	PSVhigh	NAVAlow	NAVAal	NAVAhigh	p value*		
							level	mode	interaction
**Doppler Flow Profile in the Left Ventricular Outflow Tract, n=7**									
**values at end-expiration**									
Acceleration time, ms	91 ± 13	95 ± 21	94 ± 14	98 ± 17	104 ± 26	91 ± 21	0.311	0.282	0.214
Flow period, ms	233 ± 24	236 ± 31	226 ± 27	232 ± 23	231 ± 25	234 ± 25	0.748	0.733	0.288
Maximum flow velocity, meters per second	0.9 ± 0.1	1 ± 0.2	0.8 ± 0.1	0.9 ± 0.1	1 ± 0.1	1 ± 0.1	0.499	0.623	0.252
Velocity time integral, centimeters	16 ± 3	16 ± 4	15 ± 3	16 ± 3	17 ± 3	16 ± 4	0.068	0.567	0.54
**values at end-inspiration**									
Acceleration time, ms	96 ± 13	96 ± 20	99 ± 10	98 ± 17	110 ± 25	98 ± 18	0.334	0.185	0.059
Flow period, ms	227 ± 27	237 ± 32	231 ± 22	227 ± 20	232 ± 28	238 ± 25	0.23	0.687	0.403
Maximum flow velocity, metres per second	1 ± 0.1	1 ± 0.2	1 ± 0.1	1 ± 0.1	1 ± 0.1	1 ± 0.1	0.399	0.779	0.55
Velocity time integral, centimetres	16 ± 2	17 ± 4	17 ± 3	16 ± 3	17 ± 3	16 ± 3	0.107	0.698	0.823
**inspiratory values in % of expiratory values**									
Acceleration time, %	101 ± 6	106 ± 4	108 ± 8	106 ± 5	101 ± 5	106 ± 11	0.321	0.576	0.139
Flow period, %	100 ± 2	100 ± 3	102 ± 3	98 ± 2	100 ± 3	100 ± 3	0.242	0.065	0.264
Maximum flow velocity, %	101 ± 1	103 ± 5	104 ± 2	102 ± 2	106 ± 5	110 ± 5	0.011	0.007	0.068
Velocity time integral, %	102 ± 4	108 ± 4	111 ± 7	99 ± 3	103 ± 5	105 ± 5	0.005	0.011	0.485

Addittional Table 2:

Doppler flow profiles in the left ventricular outflow tract could be obtained from seven patients. Absolute values at end-expiration did not differ between NAVA and PSV. To describe cyclic changes, the inspiratory values are given as a percentage of the corresponding expiratory value. Values are mean ± standard deviation. *p values from repeated measures ANOVA (within subject factors: ventilation mode, support level)
